# Supplementary material for: Activation of the RIG-I/MAVS Signaling Pathway during Human Adenovirus Type 3 Infection Impairs the Pro-Inflammatory Response Induced by Secondary Infection with Staphylococcus aureus
Source: Int J Mol Sci. 2024 Apr 10;25(8):4178. doi: 10.3390/ijms25084178 (PMC11049948; doi:10.3390/ijms25084178)
Supplement: Supplementary file 1 [file ijms-25-04178-s001.zip › ijms-2818772-supplementary.pdf]

Supplementary Material

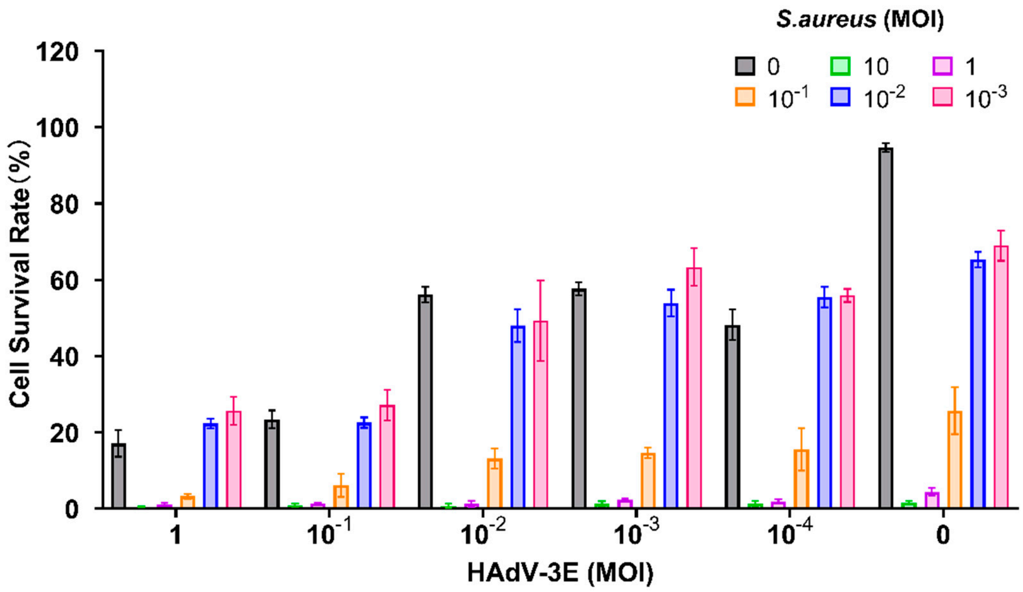

Supplementary Figures S1 Survival rate of A549 cells after infection with HAdV-3E at different MOI values for 48 hours and co-infection with *S. aureus* at different MOI values for 9 hours

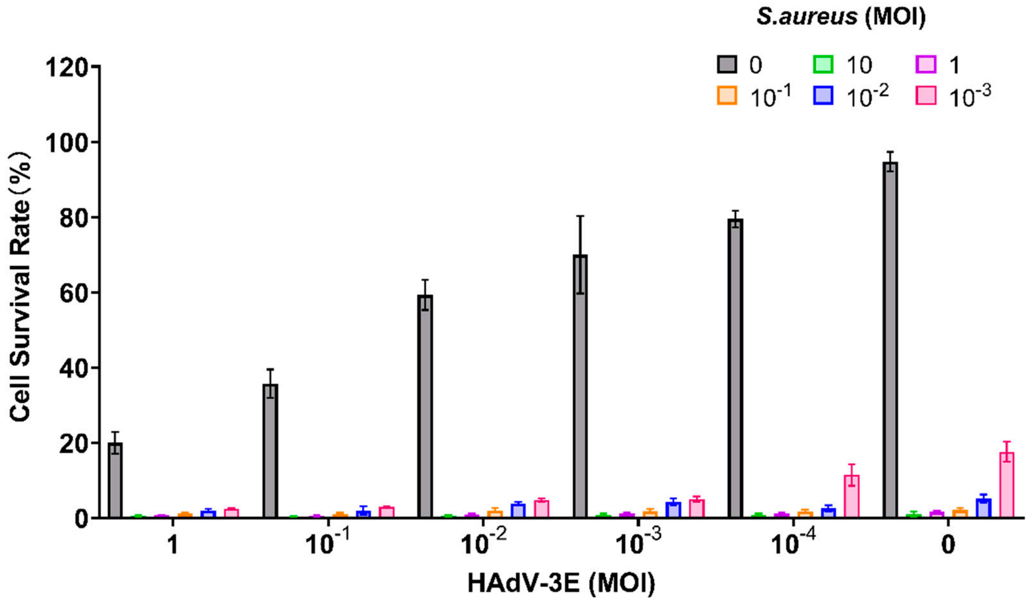

Supplementary Figures S2 Survival rate of A549 cells after infection with HAdV-3E at different MOI values for 48 hours and co-infection with *S. aureus* at different MOI values for 12 hours

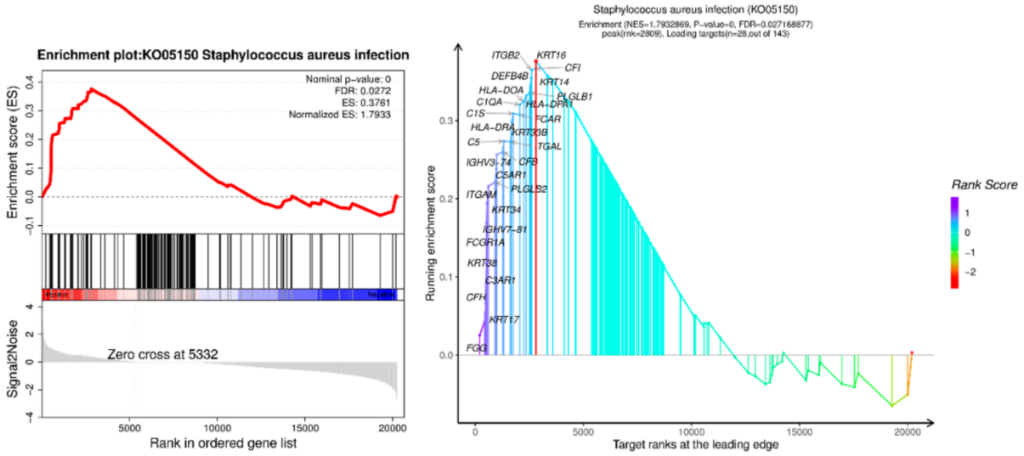

Supplementary Figures S3 A3SA_48_vs_A3SA_24 GSEA enrichment map of *Staphylococcus aureus* infection pathway

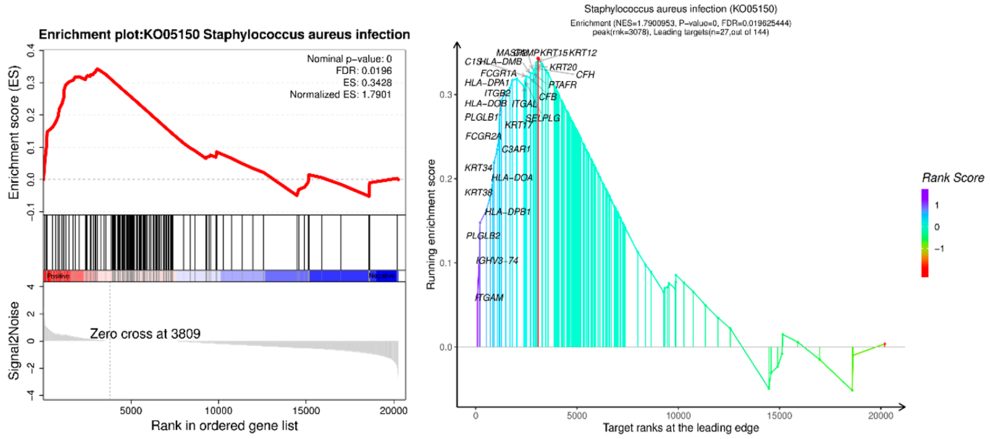

Supplementary Figures S4 A3SA_36_vs_A3SA_24 GSEA enrichment map of *Staphylococcus aureus* infection pathway

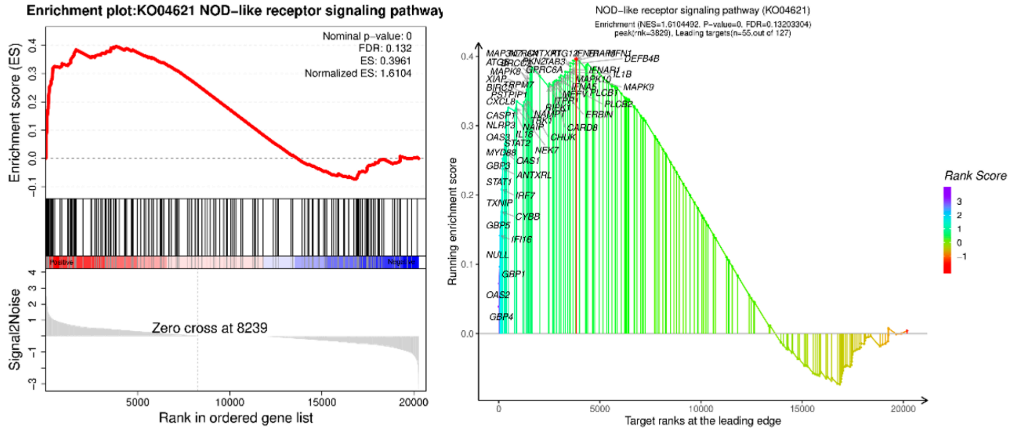

Supplementary Figures S5 A3SA_48_vs_A3SA_36 NOD-like receptor signaling pathway GSEA enrichment

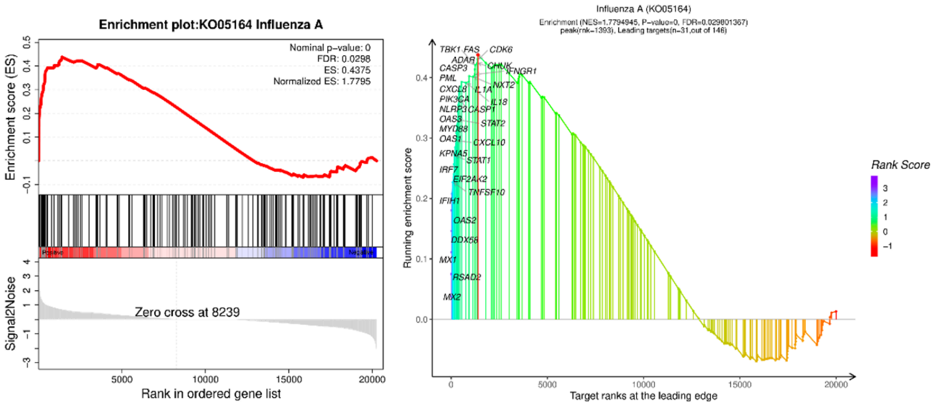

Supplementary Figures S6 A3SA_48_vs_A3SA_36 GSEA enrichment map of influenza A pathway
